# Eyesight and School Life

**Published:** 1896-12

**Authors:** 


**Eyesight and School Life.** By Simeon Snell. Pp. viii., 70. Bristol: John Wright & Co. 1895.

The author of this book well says that "day by day it becomes more evident that good vision in all walks of life is no luxury, but a necessity, and many of the avenues to employment are being closed to those who possess impaired eyesight." It is obviously of the greatest importance that every care should be taken during the period of development and of education, that delicate eyes should not be overworked, and that the requirements of study should be under conditions which are made as little deleterious as possible to health. Overworked eyes were at one time rested: now-a-days absence from school is not easily conceded; and all available artificial aid is invoked, that the student may continue his daily toil.

The little book before us is written by an ophthalmic surgeon who has paid special attention to the practical side of eye work under various conditions of occupation and of environment; and it may be recommended to the perusal of parents or of those who undertake to relieve them of the responsibility of guiding the progress of their children. There are many good illustrations of practical value; and the subject matter is expressed in simple language which requires no scientific knowledge in order that it may be appreciated and understood. We commend the book to all interested in the welfare of the young.

